# Sequence Analysis and Comparative Study of the Protein Subunits of Archaeal RNase P

**DOI:** 10.3390/biom6020022

**Published:** 2016-04-20

**Authors:** Manoj P. Samanta, Stella M. Lai, Charles J. Daniels, Venkat Gopalan

**Affiliations:** 1 Systemix Institute, Redmond, WA 98053, USA; 2 Department of Chemistry & Biochemistry, The Ohio State University, Columbus, OH 43210, USA; lai.231@osu.edu; 3 Center for RNA Biology, The Ohio State University, Columbus, OH 43210, USA; daniels.7@osu.edu; 4 Department of Microbiology, The Ohio State University, Columbus, OH 43210, USA

**Keywords:** tRNA processing, RNP evolution, RNase P, archaea, sequence analysis

## Abstract

RNase P, a ribozyme-based ribonucleoprotein (RNP) complex that catalyzes tRNA 5′-maturation, is ubiquitous in all domains of life, but the evolution of its protein components (RNase P proteins, RPPs) is not well understood. Archaeal RPPs may provide clues on how the complex evolved from an ancient ribozyme to an RNP with multiple archaeal and eukaryotic (homologous) RPPs, which are unrelated to the single bacterial RPP. Here, we analyzed the sequence and structure of archaeal RPPs from over 600 available genomes. All five RPPs are found in eight archaeal phyla, suggesting that these RPPs arose early in archaeal evolutionary history. The putative ancestral genomic loci of archaeal RPPs include genes encoding several members of ribosome, exosome, and proteasome complexes, which may indicate coevolution/coordinate regulation of RNase P with other core cellular machineries. Despite being ancient, RPPs generally lack sequence conservation compared to other universal proteins. By analyzing the relative frequency of residues at every position in the context of the high-resolution structures of each of the RPPs (either alone or as functional binary complexes), we suggest residues for mutational analysis that may help uncover structure-function relationships in RPPs.

## 1. Introduction

RNase P, a ubiquitous endonuclease responsible for tRNA 5′ maturation in all three domains of life [1,2,3,4,5,6,7], functions as one of two distinct scaffolds: either a ribozyme-based ribonucleoprotein (RNP) or an RNA-free, proteinaceous form [4,8,9]; here, we focus on the RNP. The diversity of RNase P RNP variants is exemplified by the association of a single catalytic RNase P RNA (RPR) with one, (up to) five, and (up to) ten RNase P protein (RPP) subunits in Bacteria, Archaea, and Eukarya (nuclear isoform), respectively. Where examined, all subunits in these RNPs were found essential for cellular viability [10,11,12,13].

Archaeal and eukaryotic cells share many key components of their replication, transcription, and translation machineries [14]. This relationship includes the RPPs [4,15] and other translation-related systems, including snoRNP-dependent modifications and tRNA intron processing [16]. It has also been suggested that this deep evolutionary relationship is evidence of eukaryogenesis from archaea [17]. Given that archaeal RPPs are homologous to some eukaryotic RPPs and that both suites are unrelated to the bacterial one [18,19], archaeal RNase P has been used as a tractable experimental model for biochemical and structural studies designed to uncover the basis for functional dependence of RNase P on *multiple* RPPs [20,21,22,23,24,25,26,27,28,29,30,31,32,33,34,35,36]. To gain some insight into possible predecessors that eventually led to the higher protein:RNA ratio in archaeal and eukaryotic RNase P, we examined the large number of archaeal genomes now available and used the resulting inventory of archaeal RPPs to analyze the evolution and structure of the protein subunits of archaeal RNase P.

Isolation and characterization of eukaryotic native nuclear RNase P motivated studies of archaeal RNase P. Yeast (*Saccharomyces cerevisiae*) and human nuclear RNase P comprise an RPR (~100 kDa) plus nine and ten RPPs (~15 to 100 kDa), respectively [5,6,10,37,38]. The human RPPs are called POP1, POP5, RPP14, RPP20, RPP21, RPP25, RPP29, RPP30, RPP38, and RPP40, with RPP40 lacking a homolog in yeast. Using polyclonal antisera individually raised against *Methanothermobacter thermautotrophicus* (*Mth*) RPP21, RPP29, POP5, and RPP30, which were first identified based on the corresponding yeast/human homologs, tRNA 5′ maturation activity was immunoprecipitated from a partially purified *Mth* RNase P preparation [19]. This finding inspired us and others to pursue biochemical reconstitutions of *Mth*, *Pyrococcus furiosus* (*Pfu*), *Pyrococcus horikoshii* (*Pho*), *Methanocaldococcus jannaschii* (*Mja*), and *Methanococcus maripaludis* (*Mma*) RNase P [22,24,25,26,27,35,36,39].

Our *in vitro* reconstitution studies revealed that the four archaeal RPPs function as two binary complexes: RPP21•RPP29 and POP5•RPP30 [22,25,26]. However, we found that *Pfu* RNase P assembled from RPR + RPP21•RPP29 + POP5•RPP30 was less active and displayed a lower temperature optimum than the native enzyme [26]. Extending a report that the ribosomal protein L7Ae, which exhibits ~25% sequence identity to human RPP38 [37], increases the temperature optimum of *in vitro* assembled *Pho* RNase P [39], we subsequently validated L7Ae as a *bona fide* RPP [35]. Specifically, we showed that L7Ae associates with *Mma* RNase P *in vivo* by analyzing purified native enzyme fractions with L7Ae-specific antibodies; inclusion of L7Ae also enhanced the *k*_cat_/*K*_m_ of the RPR + four RPPs by 360-fold [35]. Recently, we showed that *Pfu* L7Ae, as expected from its RNA-binding determinants, binds in the vicinity of predicted kink (K)-turn motifs in the *Pfu* RPR [40].

The last common ancestor of archaeal and eukaryotic RNase P appears to have consisted of one RPR and five different RPPs, including L7Ae. The less complex one, RPR: one RPP bacterial variant raises the question of whether “intermediate” forms of archaeal/eukaryotic RNase P with fewer RPPs than the extant set exist at the root of the archaeal tree. We report here that this scenario is unlikely based on our analysis of a large collection of archaeal genomes, and we also provide additional insights on the evolution and structure of archaeal RNase P.

## 2. Results

### 2.1. Inventory of Archaeal RPPs

We examined 678 archaeal genomes available from the National Center for Biotechnology Information (NCBI) and Integrated Microbial Genomes (IMG) databases. This set includes members of Euryarchaeota (446 genomes), Crenarchaeota (119 genomes), Thaumarchaeota (60 genomes), Nanoarchaeota (10 genomes), and newly recognized phyla Bathyarchaeota, Nanohaloarchaeota, Diapherotrites, Aenigmarchaeota, Parvarchaeota, Lokiarchaeota, and Korarchaeota (21 genomes) (Table S1). Our analysis utilized previously annotated RPPs from the UniProt database, as well as those identified in the IMG database by their matches to COG, Pfam [41], or KO domain patterns.

Prior to searching for RPPs, we first removed redundancies. The genomes from the NCBI database included several incomplete assemblies as well as multiple copies of genomes from well-studied archaeal species and subspecies. For example, the data set contained 95 Methanosarcina genomes, of which 68 were only assembled at the scaffold or contig level. We kept only one assembly for each genus, giving preference to the complete assembly wherever possible, and removed genomes without genus or species assignments (e.g., *Crenarchaeote SCGC AAA261-C22*) This process led to a non-redundant set of 127 genomes (83 Euryarchaeota, 21 Crenarchaeota, 14 Thaumarchaeota, and nine from other phyla), on which BLAST and Pfam searches for RPPs were performed. All five RPPs were found in one or more members of Euryarchaeota, Crenarchaeota, Thaumarchaeota, Nanoarchaeota, Bathyarchaeota, Nanohaloarchaeota, Diapherotrites, and Lokiarchaeota (Tables S1 and S2), which suggests that the entire set was likely present in the common ancestor of all archaea. This observation is further confirmed by analyzing putative ancestral genomic loci (see Section 2.2 below).

### 2.2. Conservation of the Genomic Neighborhoods of Archaeal RPPs

**Figure 1:**
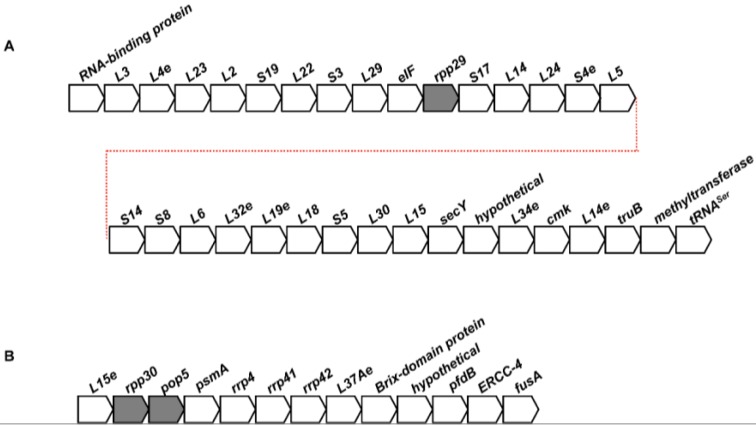
Putative ancestral loci of genes encoding RPPs. (**A**) RPP29 (**B**) POP5•RPP30.

By comparing their current neighborhoods in the non-redundant set of 127 genomes, putative ancestral loci for the RPP genes were identified (Figure 1). RPP29 belongs to a sizeable operon containing small and large subunit ribosomal proteins, as was observed previously [13]. This operon is also conserved in bacteria, albeit lacking RPP29 and eIF [13,42]. Our analysis, which used a much larger collection of archaeal genomes than any previous study, also confirms an earlier finding that POP5 and RPP30 are adjacent to one another in an operon (Figure 1) that includes genes for the proteasome and exosome [43,44], which are absent in bacteria. In the case of RPP21 (not shown), we found that its gene immediately precedes that encoding yhbY (also an RNA-binding protein), which are both then followed in some instances by ribosomal proteins; there is currently no evidence, however, that RPP21 and yhbY are co-transcribed. The genomic association of archaeal L7Ae and S28e, which has been noted previously [42], reflects coordination between members of the translation machinery.

Among the putative RPP-containing ancestral loci, the one with RPP29 is conserved to the highest extent in the archaeal genomes examined. We also note that some archaeal orders (e.g., Desulfurococcales) display complete or near-complete preservation of all RPP-containing ancestral loci while other orders (e.g., Thermoproteales) have fragmented or completely absent loci. Dynamic rearrangements of these clusters are surprising, given the essential nature of the proteins in these loci. Despite these genomic alterations, however, the nexus between RNase P, ribosome, exosome, and proteasome does appear to hold across the large collection of archaea examined.

### 2.3. Structure-Function Analyses of Archaeal RPPs

RPP structures fall within common nucleic acid-binding protein families: a zinc ribbon (RPP21), an Sm-like fold (RPP29), an RRM-like fold (POP5), and a TIM barrel (RPP30) [20,21,23,27,28,29,30,31,32,34]. Moreover, high-resolution structures of the two binary RPP complexes (RPP21•RPP29 and POP5•RPP30) identify the protein–protein interface [23,28,29,45] while high-resolution structures of L7Ae bound to RNA ligands identify the RNA–protein interface [46,47,48,49,50,51,52,53,54,55,56]. The insights below, derived from sequence and motif analyses, provide a basis for future mutagenesis efforts to help establish structure-function relationships in RPPs.

**Figure 2:**
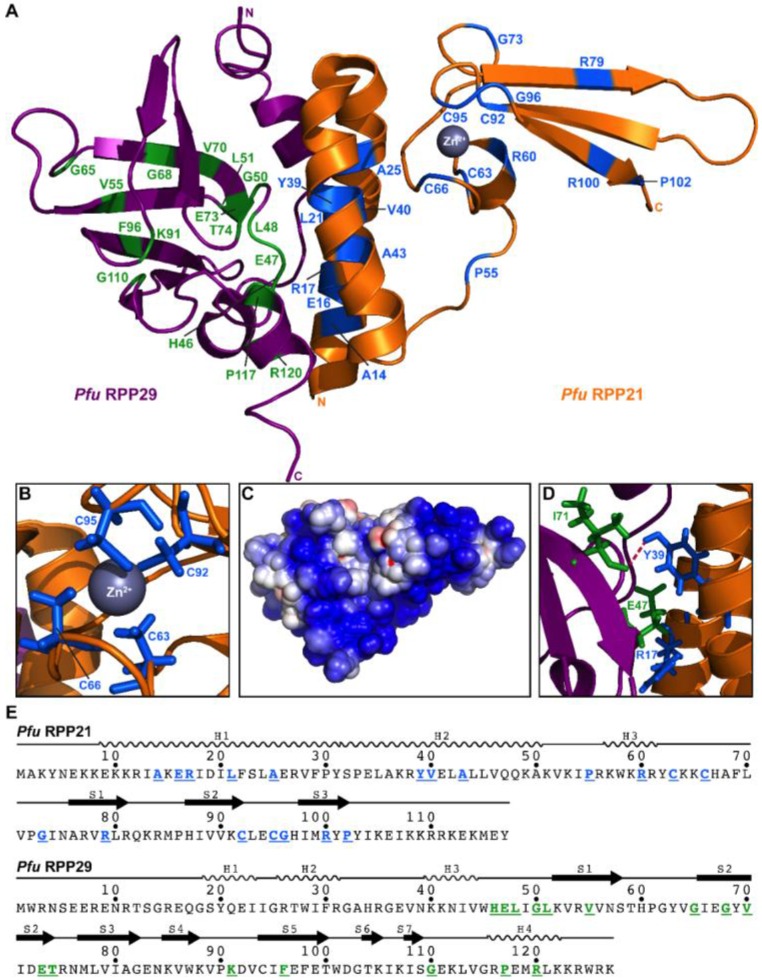
Highly conserved residues and protein–protein interactions in *Pfu* RPP21 and RPP29. (**A**) Tertiary structure of the *Pfu* RPP21•RPP29 binary complex (PDB: 2KI7 [28]). All positions with ≥80% identity from the alignment of 71 representative archaeal RPP21 and RPP29 sequences are highlighted; (**B**) Zinc-ribbon motif in RPP21 that is universally conserved; (**C**) Electrostatic potential map of the surface of the RPP21•RPP29 complex; the orientation is identical to that in panel A; (**D**) Protein–protein interactions previously identified [28]: I71 in RPP29 and Y39 in RPP21 participate in a hydrophobic interaction while E47 in RPP29 and R17 in RPP21 appear to form an intermolecular salt bridge; (**E**) Location of conserved residues (underlined and using the same color scheme as in (A)) and secondary structure elements in the sequences of RPP21 and RPP29.

**Figure 3:**
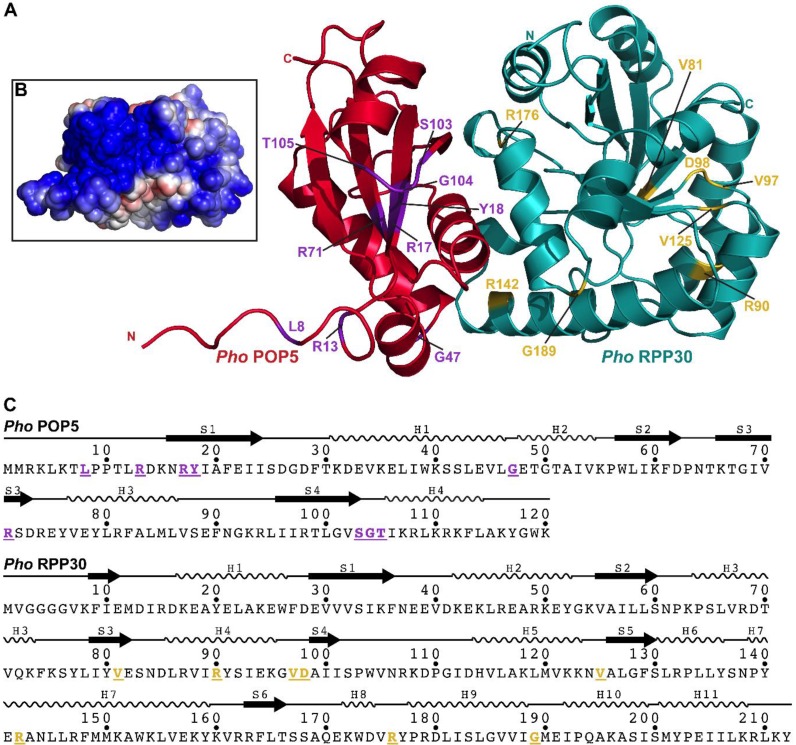
Highly conserved residues in *Pho* POP5 and RPP30. (**A**) Tertiary structure of the *Pho* POP5•RPP30 binary complex (PDB: 2CZV [23]). All positions with ≥80% or ≥75% identity from the alignment of 71 representative archaeal POP5 and RPP30 sequences, respectively, are highlighted; (**B**) Electrostatic potential map of the surface of the POP5•RPP30 complex; the orientation is identical to that in panel A; (**C**) Location of conserved residues (underlined and using the same color scheme as in (A)) and secondary structure elements in the sequences of POP5 and RPP30.

**Figure 4:**
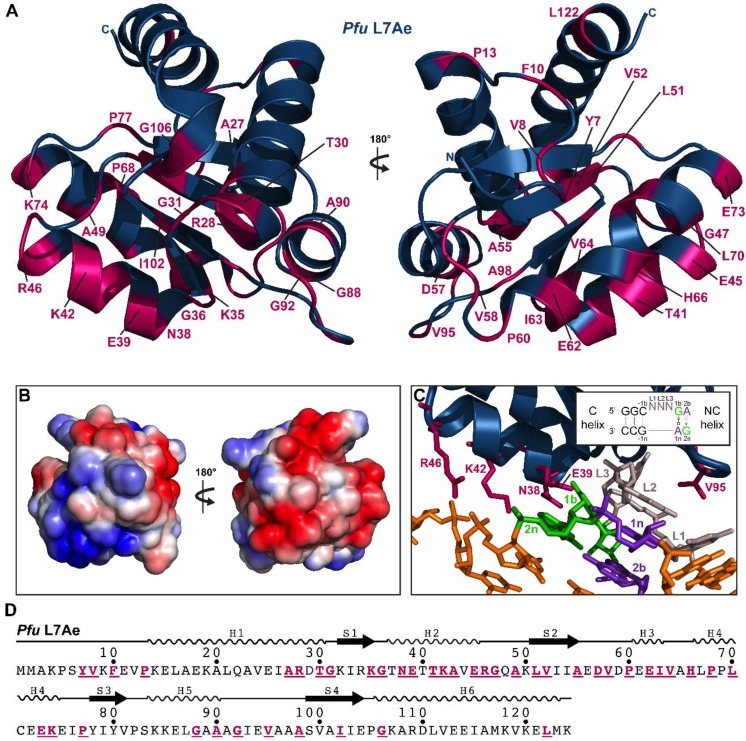
Highly conserved residues and RNA–protein interactions in *Pfu* L7Ae. (**A**) Tertiary structure of *Pfu* L7Ae [PDB: 3NVI [55]]. All positions with ≥80% identity from the alignment of 71 representative archaeal L7Ae sequences are highlighted; (**B**) Electrostatic potential map of the surface of *Pfu* L7Ae; the orientations are identical to those in panel A; (**C**) Binding interface between *Pfu* L7Ae and a box C/D RNA [PDB: 3NVI [55]]. Residues involved in interactions with the RNA ligand are highlighted. Inset shows a standard K-turn motif with its canonical (C) and non-canonical (NC) helices [49]; the two sheared G•A base pairs as well as the three-nucleotide bulge have been color-coded in both the inset and the main panel; (**D**) Location of conserved residues (underlined and using the same color scheme as in (A)) and secondary structure elements in the sequence of L7Ae.

Our initial goal was to generate sequence alignments for each of the five RPPs. First, we identified genomes that encode RPP21, RPP29, POP5, and RPP30. The list of genomes was again filtered to remove duplicates and to obtain a final set with each RPP having less than 80% sequence identity with its nearest sequence neighbor (see Supplementary). To the resultant collection, we manually added *Pho* and *Bathyarchaeota archaeon BA1* (or *strain BA2* in the case of L7Ae, as it was not detected in the draft genome sequence of strain *BA1*) to yield a final set of 71 genomes (Table S3), which was then used to generate sequence alignments and logo maps (Figure S1). An arbitrary cut-off of 80% identity (except for RPP30, where 75% was used) in the final alignments was used to highlight conserved residues and dissect their possible functional significance based on the high-resolution structures of the RPPs, either alone or as binary complexes: the NMR solution structure of *Pfu* RPP21•RPP29 (Figure 2; [28]) and the crystal structures of *Pho* POP5•RPP30 (Figure 3; [23]) and *Pfu* L7Ae (Figure 4; [55]).

In addition, we used RPP21, RPP29, POP5, and RPP30 from the set of 71 genomes as well as their homologs from *Saccharomyces cerevisiae* [10], *Dictyostelium discoideum* [57,58,59]*,* *Xenopus laevis*, and *Homo sapiens* [38] as input for the web-based Multiple EM for Motif Elicitation (MEME) sequence analysis tool [60,61] to uncover ungapped motifs common to archaeal and eukaryotic RPPs; L7Ae was excluded from MEME profiling given the extensive structural studies and previous analyses (see 47 and references therein). The MEME search parameters used for each RPP family were zero to eight motifs of 10–30 amino acids long. We integrated this information along with insights from Pfam alignments when considering the significance of conserved residues (Table S4).

#### 2.3.1. RPP21

The zinc ribbon in the C-terminal domain of RPP21 is a motif found in other nucleic acid-binding proteins (20,30) and comprises a β-hairpin and four invariant cysteine residues (C63, C66, C92, and C95). Indeed, C63S/C66S and C92S/C95S are functionally defective as assessed by reconstitution assays of *Pho* RNase P (34). G73 and G96 are located in loops and likely ensure that the β-hairpin of the zinc ribbon motif folds correctly to enable zinc coordination by C63, C66, C92, and C95. Additionally conserved are R79 and R100, which are located in an electropositive surface (Figure 3C) and are likely involved in interactions with either the RPR or the pre-tRNA. Guided by the finding that human RPP21 binds pre-tRNA [62], we previously proposed that this positively charged surface in archaeal RPP21 similarly mediates interactions with the pre-tRNA substrate [28]. Although mutating these arginines does, in fact, lead to a 2-fold (R79A) and a near-complete loss (R100A) of *Pho* RNase P activity (34), the basis for the decrease in activity has not been investigated. In addition to RNA binding assays, introduction of affinity cleavage agents [40] at or near R79 and R100 would help uncover whether the ligand for RPP21 is the RPR or the pre-tRNA.

MEME analysis indicates that three out of eight possible motifs among archaeal RPP21 are shared between eukaryotes and archaea. Two of these motifs are located in tandem and include the four cysteines and two arginines [C(x)_2_C(x)_6_G(x)_5_R and C(x)_2_C(x)_4_R], indicating the broad functional importance of the zinc-ribbon motif. Pfam’s seed alignment of 291 archaeal and eukaryotic RPP21 homologs (pfam04032) confirms near-universal conservation of both cysteine motifs but not of the arginines; the latter residues are substituted with lysine at high frequency. A recent study that examined an alignment of eukaryotic RPP21 homologs also observed conservation of these cysteine motifs, though the motif sequences were slightly different [8].

#### 2.3.2. RPP29

The most prominent feature of RPP29 is a twisted barrel of seven antiparallel β-strands [21,30,31,32], whose conformation is maintained by a hydrophobic core (L48, L51, V55, V70) and by highly conserved glycines in the intervening loops (G50, G65, G110) and strand S2 (G68) [30]. Further stabilizing the structure of the twisted barrel of β-strands is the intramolecular salt bridge formed between universally conserved residues E73 and K91 (Figure S1A) [21,30]. The loop connecting strands S2 and S3 includes conserved residue T74, which likely participates in RNA–protein interactions through hydrogen bonding with either the RPR or pre-tRNA; in fact, the E73–K91 interaction may help position both T74 and F96 for RNA binding.

Of the three motifs that MEME detected as conserved between archaeal and eukaryotic RPP29, one includes G68, E73, and T74 while another covers K91 and F96, implicating these residues and their associated interactions as significant for both archaeal and eukaryotic RNase P function. Pfam’s seed alignment of 233 archaeal and eukaryotic RPP29 homologs (pfam01868) also confirms the near-universal conservation of the GX_4_ET motif and, to some extent, the KX_5_F arrangement.

#### 2.3.3. RPP21•RPP29

Because RPP21 functions together with RPP29, it is not unexpected that a large surface in RPP21 is devoted to interactions with RPP29. Two long N-terminal α-helices are held together by hydrophobic interactions that include A14, V40, and a pocket formed in part by L21 (universally conserved), Y39, and A43 (Figure S2B) [20,34]. These conserved residues, along with others in the helical domain (E16, R17, and A25), likely play important roles in stabilizing a RPP21 structure that favors interactions with RPP29. In fact, some of the largest backbone amide chemical shift perturbations in RPP21 that were observed by NMR [28] upon RPP29 binding belong to residues clustered in this helical bundle, including L21 and A25, which are conserved in both archaeal and eukaryotic RPP21 [8,28]. Additionally, a *Pfu* RPP21 A14V mutant was found to bind RPP29 three times weaker than the wild-type RPP21, and no new amide signals, in contrast with the wildtype, were observed upon binary complex formation [28]. This result indicates that an A14V mutation interferes with the ability of RPP21 to undergo binding-mediated folding.

Residues E16 and R17 in RPP21 form prominent intermolecular salt bridges with residues H46 and E47 in RPP29, respectively (the latter interaction is shown in Figure 2D). Additionally, the side-chain hydroxyl of Y39 in RPP21 participates in a polar contact with the backbone carbonyl of I71 in RPP29 (Figure 2D). Since RPP21 mutants R17A and Y39A showed a three-fold decrease in activity compared to wild-type *Pho* RNase P [34], these protein–protein interactions are clearly crucial for function of the binary complex. While R38 in RPP21 also forms an intermolecular salt bridge with D72 in RPP29, these two residues did not meet the 80% threshold that we used. Additionally, this intermolecular contact is not universally conserved even within archaea and is entirely absent in eukaryotes. In fact, there are structural differences in RPP29 between even closely related organisms like *Pfu* and *Pho*. Nevertheless, such differences only heighten the importance of the few conserved positions between archaea and eukaryotes (e.g., R17, L21, A25, and Y39 in RPP21) for strong protein–protein interactions.

#### 2.3.4. POP5

Despite weak sequence homology, archaeal POP5 is structurally similar to the bacterial RPP [23,27]. In both instances, β-strands form a central cleft, which in the bacterial RPP is believed to engage the 5′ leader of the pre-tRNA substrate through stacking (Phe or Tyr) and electrostatic (Arg/Lys) interactions [63,64]. Highly conserved residues Y18, R71, S103, and T105 may help POP5 accomplish a similar role. Moreover, depending on the curvature of the 5′ leader of the pre-tRNA, it is possible that R17 in POP5 could participate in contacts with the phosphate backbone. Unlike in the bacterial RPP, the putative binding pocket in POP5 is partially obscured by helix H4; however, S103 and T105 in the immediately preceding loop may serve as a hinge to displace the helix, thereby enabling the interaction of cleft residues Y18 and R71 with the 5′ leader of pre-tRNA [27]. The conservation of G104 may reflect steric considerations related to RNA binding.

MEME analysis indicates that while only two out of eight possible motifs are shared between archaeal and eukaryotic POP5, the two motifs include R71 as well as S103, G104, and T105. Moreover, a Pfam seed alignment of 220 archaeal and eukaryotic POP5 sequences (pfam01900) also identifies R17, Y18, R71, S103, G104, and T105 as near-universally conserved.

#### 2.3.5. RPP30

Of the RPPs examined, RPP30 shows the least conservation—only five out of 212 residues in *Pho* RPP30 meet the 80% sequence identity threshold, but this number increases to 11 when we use a 75% threshold. Upon mutation in *Pho* RNase P, R90A and R176A exhibit two- and three-fold decreases in activity, respectively, indicating their importance for function [33]. However, the large distance between R90 and R176 in the tertiary structure makes it somewhat unlikely that they interact with the same RNA ligand. Additional studies are clearly needed to address this aspect.

MEME analysis indicates that while only one out of eight possible motifs is conserved between archaeal and eukaryotic RPP30, this motif includes R176 and G189. An alignment of 40 archaeal and eukaryotic RPP30 sequences in Pfam (pfam01876) confirms the conservation of R176.

#### 2.3.6. POP5•RPP30

Although both the crystal structure of *Pho* POP5•RPP30 (Figure 3) and an NMR study of *Pfu* POP5•RPP30 reveal hydrophobic contacts between POP5 and RPP30 [23,45], the suite of interactions that dictates this interface shows weak conservation (below 50% identity). Thus, variability in the POP5–RPP30 interface across archaeal species seems likely.

#### 2.3.7. L7Ae

L7Ae belongs to a family of proteins that specifically recognizes and binds K-turns, which are widespread RNA structural motifs that cause an axial bend in RNA helices. The standard K-turn configuration consists of a 5′ canonical (C) helix with Watson-Crick base pairs, a three-nucleotide bulge, and a 3′ non-canonical (NC) helix with two consecutive *trans* sugar-edge•Hoogsteen sheared G•A base pairs (see Figure 4C, inset). L7Ae, comprised of a central four-stranded β-sheet surrounded by five α-helices, docks in the major groove and stabilizes the K-turn. NMR and crystallographic studies of L7Ae (and its homologs), both alone and bound to K-turn-containing RNA ligands (including rRNA, C/D or H/ACA snoRNAs), highlight the specific recognition of K-turns by highly conserved regions in L7Ae [46,47,50,51,54,55,56]. A universally conserved NEXXK motif in H2 utilizes side-chain and backbone contacts to hydrogen bond to G1b and G2n in the NC helix of the K-turn (Figure 4C). Mutating the G•A base pairs results in loss of L7Ae binding and RNP assembly [48,52] while collectively mutating the key residues of the NEXXK motif results in loss of function [35]. Moreover, R46 and V95 (conserved ≥97%) in L7Ae also play a role in RNA recognition (Figure 4C; [40,46,47,56]).

#### 2.3.8. General Remarks

Our sequence analyses identified residues conserved in only archaea (Table S4) as well as those conserved in both archaeal and eukaryotic RPPs (not shown). These conserved positions may be critical for (i) maintaining RPP structure; (ii) enabling protein–protein interactions to form binary RPP complexes; (iii) mediating RPP-RPR contacts; and (iv) promoting pre-tRNA binding. Where possible, we have parsed such contributions (see above). Despite some preliminary mutagenesis results, pre-tRNA/RPR binding and pre-tRNA cleavage assays are needed to gain insights into which of the four roles are fulfilled by specific residues in the RPPs. Conserved arginines and lysines, which are expected to contribute to RPR/pre-tRNA binding, are obvious prospects as targets for mutagenesis. Other residues, such as those in the putative RNA-binding cleft of POP5 (Y17, R71, S103, T105), also warrant a closer look.

An unexpectedly large number of glycines are highly conserved in the RPPs. The ability of glycine to adopt a wide range of φ and ψ angles makes it well suited for turns where other residues with side chains are less favored. For example, G47 in POP5 is universally conserved, likely due to its key role in facilitating the sharp turn between α-helices H1 and H2 (Figure 3A). In some other instances, however, it appears that the absence of a side chain may prove necessary to allow for RNA binding to neighboring residues, as exemplified by G104 in POP5. These postulates are testable.

## 3. Discussion

### 3.1. Divergence of Bacterial and Archaeal RNase P

Given the structural and functional similarities of the RPR in all three domains of life [3,18,65], it is likely that an RPR was present in the last universal common ancestor or progenote. Our analysis here suggests that the archaeal RPR became associated with five RPPs at a very early stage. Since the bacterial RNase P uses a single RPP to facilitate RPR catalysis in contrast to its multi-RPP archaeal/eukaryotic relatives, it appears that these numerous RNP variants of RNase P reflect a plasticity in the make-up of RNase P while retaining the ability to accomplish the same function (*i.e*., convergent evolution). This claim is supported by the finding that, while dissimilar in amino acid sequence, POP5 and the bacterial RPP share a similar tertiary fold [23,27].

Consistent with our postulate above, we predict that new variants of archaeal RNase P will be uncovered as additional phyla in unique niches are studied. Indeed, in our analyses, we found instances where the genes for some but not all RPPs are present (e.g., *Thermoplasma acidophilum*, *Nanoarchaeota* *Nst1*). It is difficult to conclude whether these instances reflect a smaller suite of RPPs or if the “missing” RPPs have diverged extensively, thereby escaping detection by sequence similarity searches. However, at least in a few cases, the change in RPP number may reflect a corresponding change in the RPR. For example, the RPR, which typically comprises a specificity and a catalytic domain, is somewhat smaller in Thermoproteaceae (phylum Crenarchaeota) due to an abbreviated specificity domain [66]. Thermoproteaceae also lack RPP21 [66], a finding consistent with our observation that RPP21•RPP29 binds to the RPR’s specificity domain and increases its apparent substrate affinity by 16-fold [22,25,26,28]. In the case of Nst1, a parasitic nanoarchaeon, the RPR is also smaller, and POP5 appears to be absent (only RPP21, RPP29, RPP30, and L7Ae were identified) [67]. Since POP5•RPP30 binds to the RPR’s catalytic domain and enhances the RPR’s rate of cleavage by 60-fold [22,25,26,28], it is possible that the catalytic domain in the *Nst1* RPR has undergone remodeling. Once these computationally predicted thematic variations in the make-up of archaeal RNase P are experimentally validated, it would be beneficial to investigate whether such rewiring of the RNase P holoenzyme (through changes to the RPR and/or RPPs) reflects either coevolutionary adaptations to a specific suite of substrates (pre-tRNAs or otherwise) and or the need to network with other macromolecular assemblies.

### 3.2. Coordinate Regulation of RNase P and Other Cellular Machineries

The presence of L7Ae in archaeal RNase P, snoRNPs, and the ribosome, which are all macromolecular machines involved in some aspect of translation, suggests coordinate regulation of these complexes. The expectation for such crosstalk is heightened by the presence of RPP29 in an operon encoding ribosomal proteins. In fact, this appears to be a recurring theme in all three domains of life: bacterial RPP is co-transcribed with the ribosomal protein L34, and yeast RNase P has been implicated in the processing of antisense RNAs from genes that encode ribosomal proteins [68,69]. The exact mechanism by which RNase P activity is coupled to expression of ribosomal proteins and other central cellular machineries offers prospects for future studies.

## 4. Conclusions

Based on our analysis of an extensive collection of archaeal genomes, we propose that the last common ancestor of archaeal and eukaryotic RNase P consisted of one RPR and five RPPs–RPP21, RPP29, POP5, RPP30, and L7Ae. Moreover, by examining sequence conservation in the context of the high-resolution structures, we recommend residues for mutational analysis to help uncover structure-function relationships in these RPPs.

## Supplementary Materials

The following are available online at http://www.mdpi.com/2218-273X/6/2/22/s1.

## Abbreviations

The following abbreviations are used in this manuscript:

MEME	Multiple EM for Motif Elucidation
NCBI	National Center for Biotechnology Information
RPP	RNase P protein
RPR	RNase P RNA
pre-tRNA	precursor tRNA
tRNA	transfer RNA
