# Supplementary material for: Sequence Analysis and Comparative Study of the Protein Subunits of Archaeal RNase P
Source: Biomolecules. 2016 Apr 20;6(2):22. doi: 10.3390/biom6020022 (PMC4919917; doi:10.3390/biom6020022)
Supplement: Supplementary file 1 [file biomolecules-06-00022-s001.zip › biomolecules-123063-supplementary-publish/biomolecules-123063-suppl.-final.pdf]

Supplementary Materials: Sequence Analysis and Comparative Study of the Protein Subunits of Archaeal RNase P

Manoj P. Samanta, Stella M. Lai, Charles J. Daniels and Venkat Gopalan

Materials and Methods

1. Online databases

Archaeal genomes were downloaded from the NCBI database (ftp://ftp.ncbi.nlm.nih.gov/genomes/). Web-based tools provided by the JGI (Joint Genome Institute) IMG (Integrated Microbial Genomes) database (http://img.jgi.doe.gov) were used for genome analysis.

2. Identification of archaeal RPPs

We first compiled a reduced list of genomes that drew from the entire set of 678 genomes downloaded from the NCBI database. Genomes without genus or species assignments (e.g., *Crenarchaeote SCGC AAA261-C22*) were removed, and only one genome per genus was kept (with preference given to fully assembled genomes wherever possible). This process yielded a reduced set of 127 genomes (highlighted in yellow in Table S1B). BLAST searches (tblastn, cutoff 1e-5) for all five RPPs were conducted against this reduced set of genomes. Previously annotated proteins from the Uniprot database (http://www.uniprot.org/) as well as those identified in the IMG database based on COG, Pfam, and KO domain pattern matches were also used in our final compilation.

3. Alignment of RPPs

To generate sequence alignments for each of the four RPPs, web-based tools from the IMG database were used to obtain a shortlist of sequences. Genomes from the IMG database were queried for the presence of RPP21, RPP29, POP5, and RPP30 using the COGs, Pfams, and KOs for each protein (see below). Duplicates were removed using the IMG command "drop=d". Overrepresentation of closely related sequences was addressed by sequential use of the USEARCH algorithms cluster_fast and uclust, with the limit of relatedness set to < 80% identity.

Protein	COG	Pfam	KO
POP5	COG1369	pfam01900	K03537
RPP21	COG2023	pfam04032	K03540
RPP29	COG1588	pfam01868	K03538
RPP30	COG1603	pfam01876	K03539

A total of 69 genomes met these stringent requirements. Two additional genomes, *Pyrococcus horikoshii* and *Bathyarchaeota archaeon BA1*, were manually added to comprise the final set (Table S3). RPP sequences were then aligned using the MUSCLE program (http://www.drive5.com/muscle/) [1] and graphically represented using the web-based Berkeley WebLogo application (http://weblogo.berkeley.edu/logo.cgi) [2].

To depict the highly conserved residues in the high-resolution structures of *Pyrococcus furiosus* RPP21•RPP29 and *Pyrococcus horikoshii* POP5•RPP30, the relative frequency of every amino acid at each position in the protein sequence was calculated. For example, if there were 60 arginines found at a particular position out of 71 sequences, an 84.5% sequence identity for arginine was noted.

4. Putative Ancestral Loci of RPP Genes

The 127 non-redundant "reduced set" of genomes was used to find genomic loci containing RPP genes, which were then compared to find the longest common contiguous locus supported by at least two phyla. We defined a locus as a contiguous set of genes with the same transcriptional polarity.

For RPP29, *Methanothermobacter marburgensis (strain DSM 2133)* from Euryarchaeota and *Desulfurococcus fermentans Z-1312 (strain DSM 16532)* from Crenarchaeota provide support for the longest

common contiguous locus, as shown in Figure 1A. The inclusion of eIF is supported by *Methanothermus fervidus V24S (strain DSM 2088)* from Euryarchaeota and *Nanoarchaeota archaeon SCGC AAA011-L22* from Nanoarchaeota.

For POP5 and RPP30, *Methanothermobacter marburgensis (strain DSM 2133)* from Euryarchaeota and *Thermogladius cellulolyticus (strain 1633)* from Crenarchaeota provide support for the longest common contiguous locus, as shown in Figure 1B.

For RPP21, Methanothermus fervidus V24S (strain DSM 2088) from Euryarchaeota and Desulfurococcus fermentans Z-1312 (strain DSM 16532) from Crenarchaeota provide support for the longest common contiguous locus (not shown): rpp21, yhbY, S19e, TFAR19 protein, L39e, L31e, eIF6, L18a, pfdA, and ftsY.

Table S1. Archaeal genomes available from the NCBI database. (A) Distribution of RPPs in different archaeal phyla. (B) Complete list of archaeal genomes. Genomes highlighted in yellow are part of the reduced list of 127 genomes used for loci analysis.

(See MSamanta_Supplementary_Tables.xls)

Table S2. Tabulation of RPPs in each archaeal phylum**.** (A) Euryarchaeota (B) Crenarchaeota (C) Thaumarchaeota (D) Other archaeal phyla. Y, present; N, absent.

(See MSamanta_Supplementary_Tables.xls)

Table S3. List of 71 archaeal genomes whose RPPs were used in the sequence alignments.

(See MSamanta_Supplementary_Tables.xls)

Table S4. Conserved residues with identity scores.

RPP21		RPP29		POP5		RPP30		L7Ae	
Residue	% Identity	Residue	% Identity	Residue	% Identity	Residue	% Identity	Residue	% Identity
A14	84	H46	88	L8	84	V81	78	Y7	87
E16	80	E47	87	R13	88	G84	76	V8	87
R17	92	L48	83	R17	95	R90	97	F10	83
L21	100	G50	98	Y18	98	V97	81	P13	97
A25	97	L51	94	G47	100	D98	80	A27	84
Y39	90	V55	81	R71	84	L100	78	R28	81
V40	90	G65	83	S103	90	A120	76	T30	84
A43	84	G68	100	G104	97	V125	80	G31	91
R51	80	V70	80	T105	90	R142	84	K35	98
P55	81	E73	100			P162	80	G36	100
R60	81	T74	92			R176	76	N38	100
C63	100	K91	100			G189	77	E39	100
C66	100	F96	88					T41	100
G73	92	V106	84					K42	100
R79	98	G110	100					A43	80
C92	100	P117	94					E45	100
C95	100	R120	98					R46	97
G96	92							G47	85
R100	91							A49	88
P102	80							L51	98
								V52	84
								A55	83
								D57	98
								V58	87
								P60	100
								E62	100
								I63	88
								V64	88
								H66	100
								P68	92
								L70	88
								E73	98
								K74	97
								P77	87
								G88	100
								A90	97
								G92	97
								V95	100
								A98	85
								I102	88
								G106	95
								L122	90

Conserved residues in each of the five RPPs. The ones highlighted in red indicate conserved residues that were not included in our analysis due to their absence in the sequences of *Pfu* RPP21 or RPP29 or L7Ae or *Pho* POP5 or RPP30.

A. RPP21

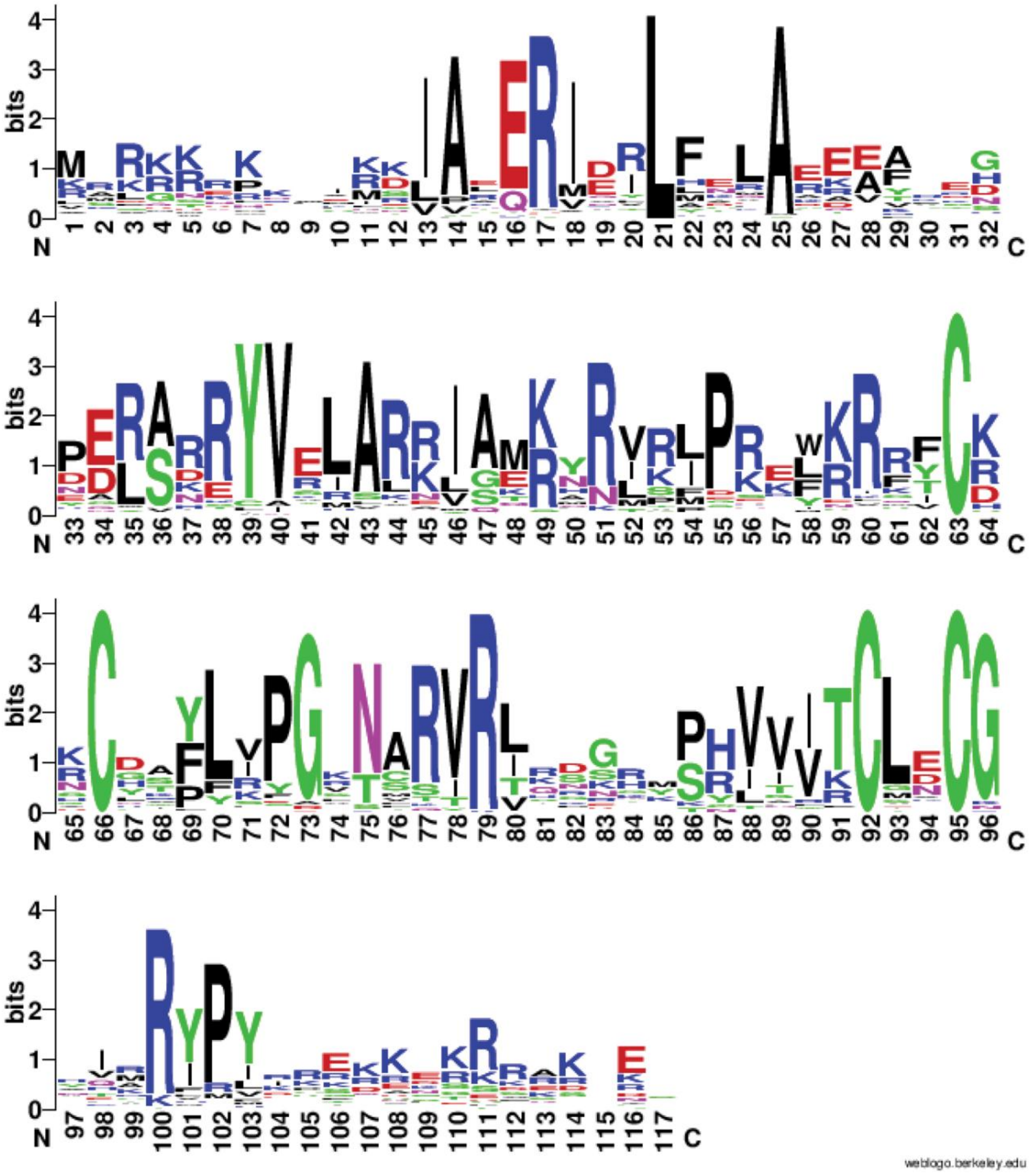

Figure S1. *Cont*.

B. RPP29

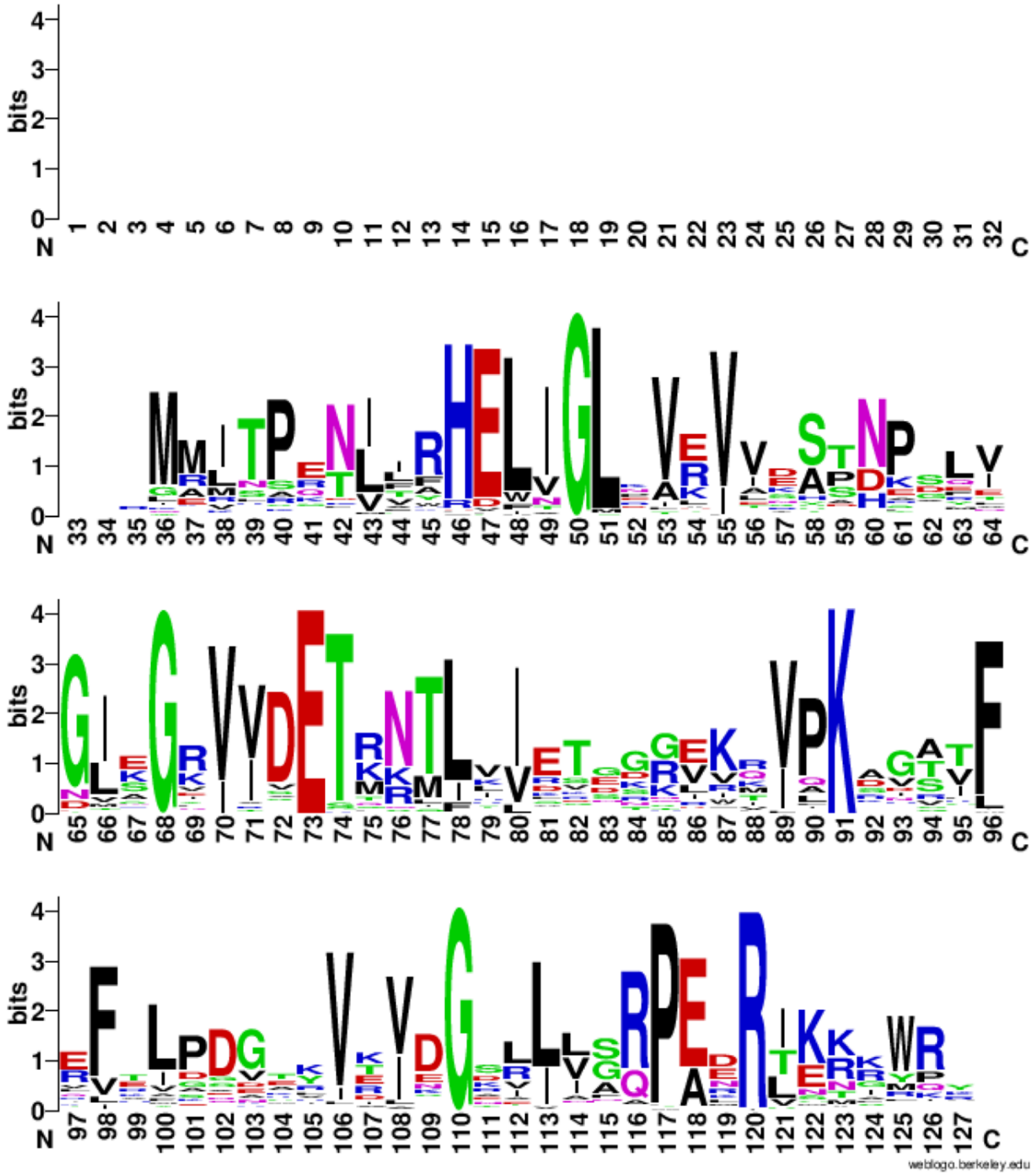

Figure S1. *Cont*.

C. POP5

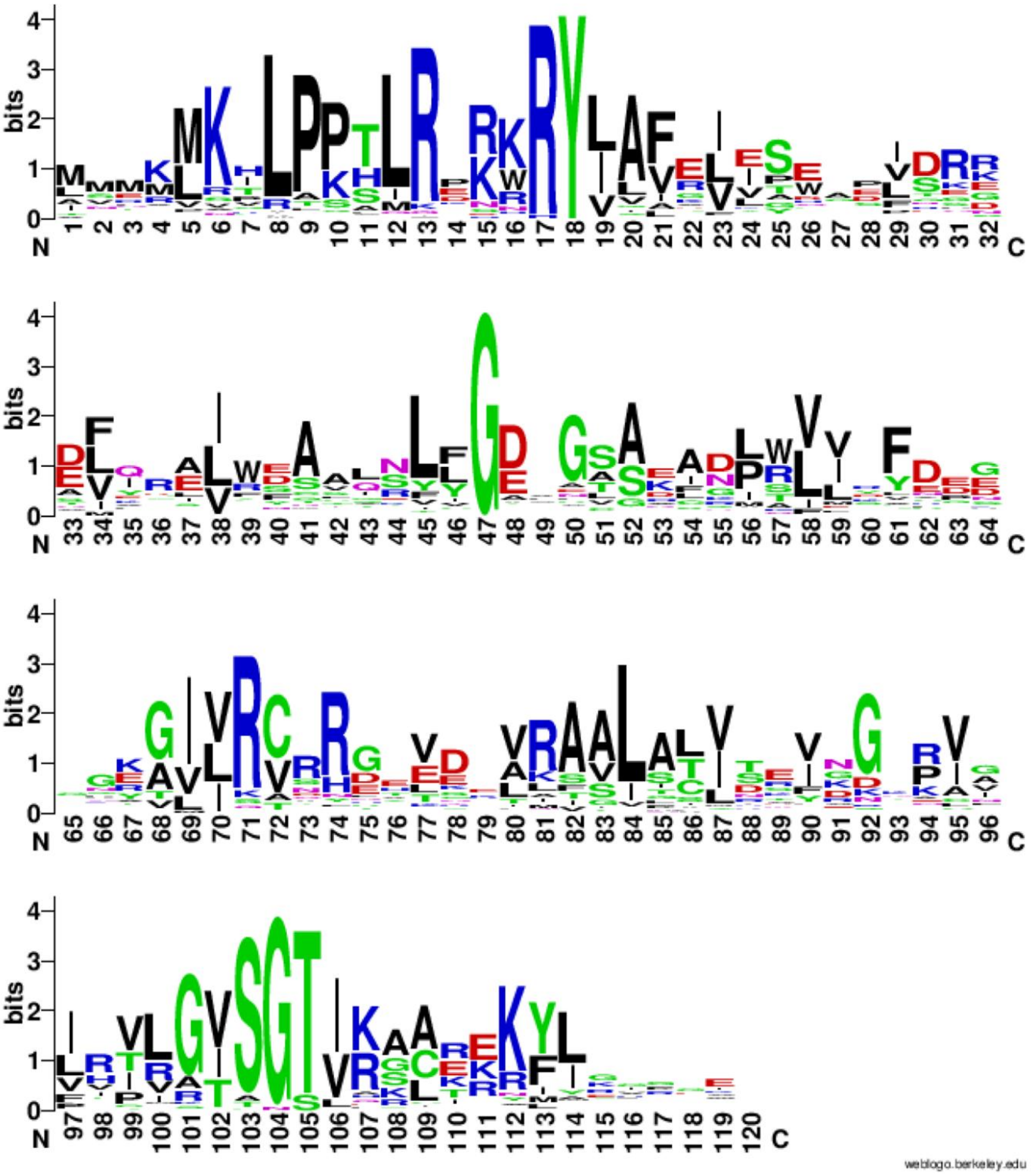

Figure S1. *Cont*.

D. RPP30

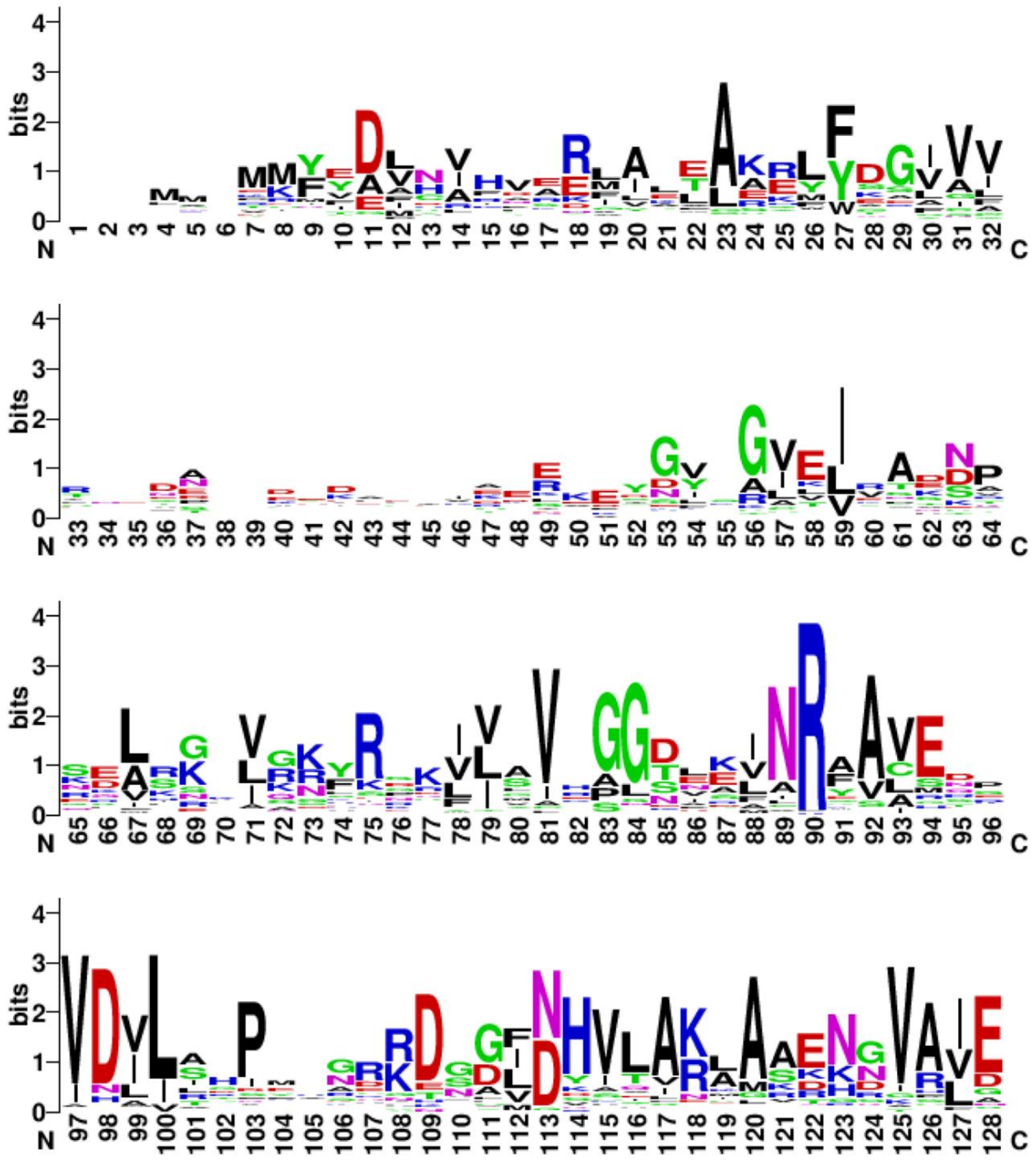

Figure S1. *Cont***.**

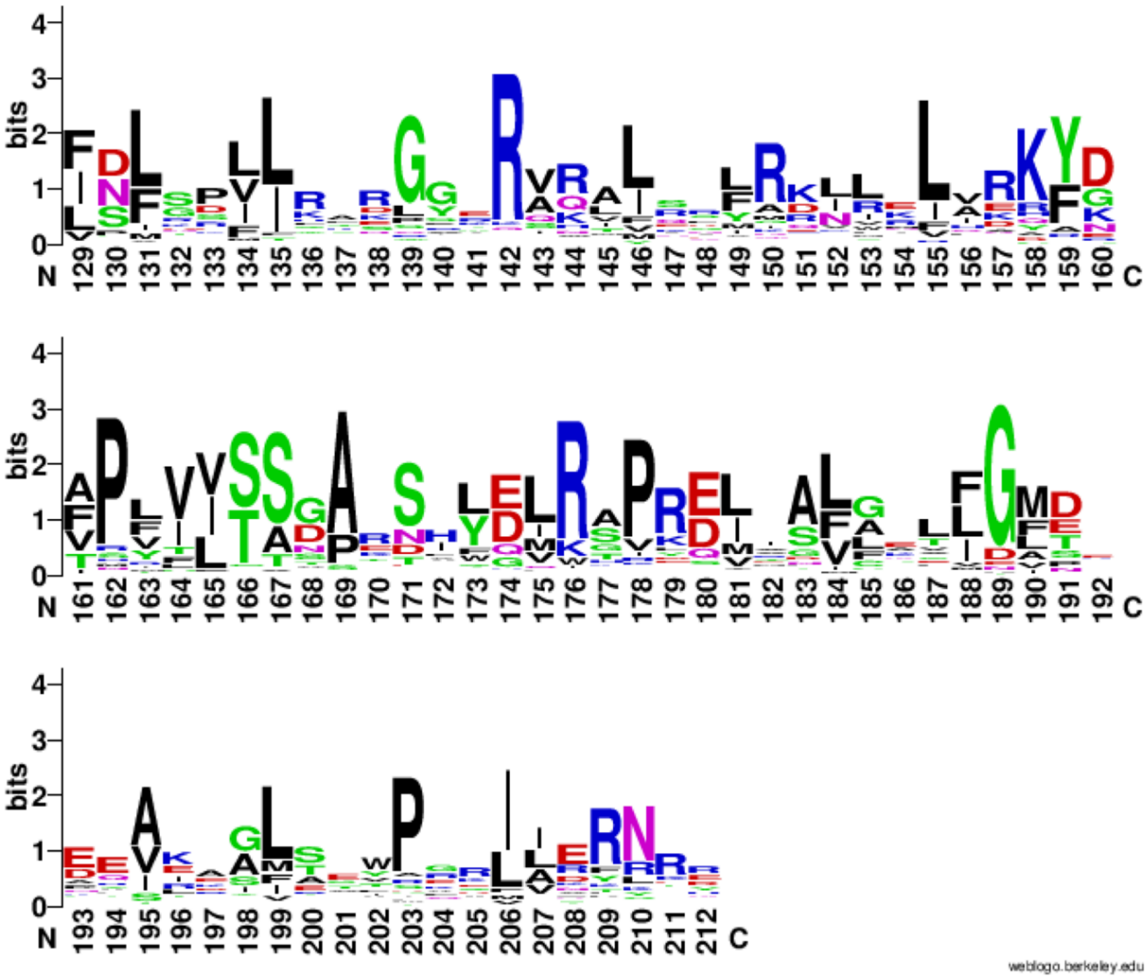

Figure S1. *Cont*.

E. L7Ae

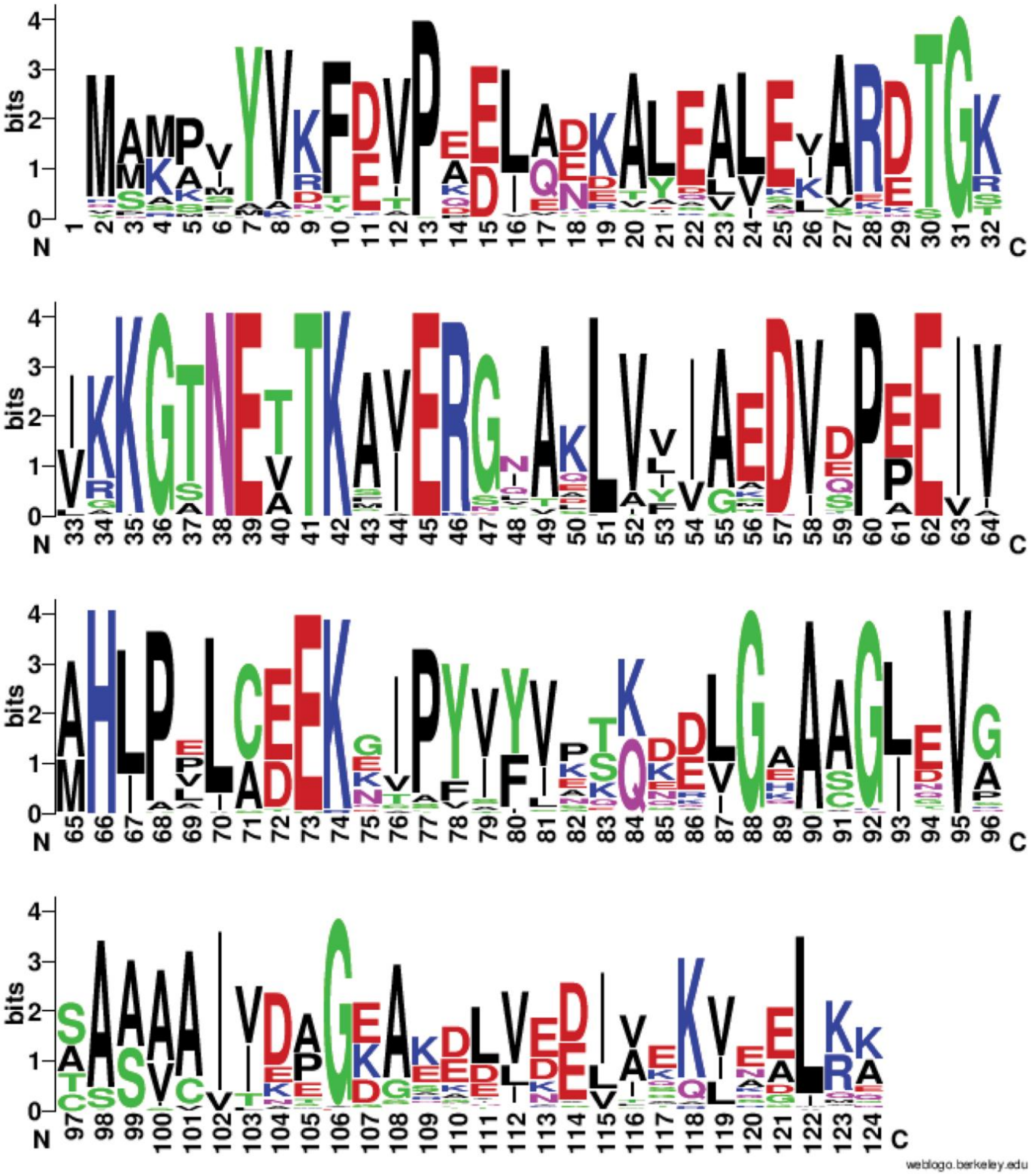

Figure S1. Conservation of amino acid identity in the five RPPs across 71 archaeal genomes, as depicted using the web-based Berkeley WebLogo application (http://weblogo.berkeley.edu/logo.cgi) [2]. (A) RPP21 (B) RPP29 (C) POP5 (D) RPP30 (E) L7Ae.

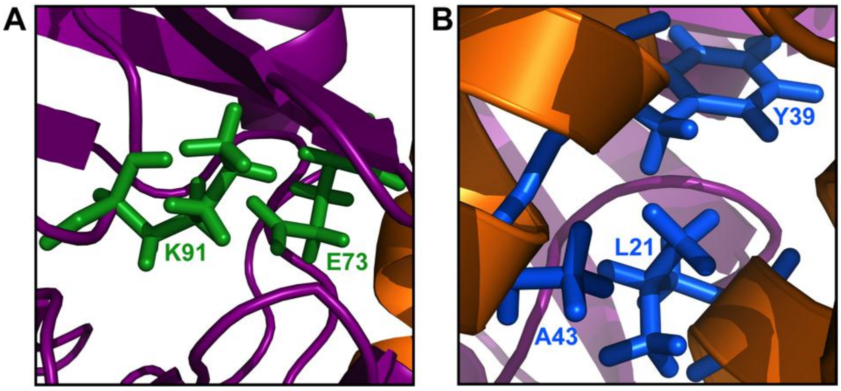

Figure S2. (A) Intramolecular salt bridge in the interior of *Pfu* RPP29 that helps form a hydrogen bond network connecting strand S2 and helix H4 [3]. (B) Highly conserved residues that form part of the hydrophobic core which anchors the two main helices of *Pfu* RPP21 [4].

References

1. Edgar, R.C. MUSCLE: Multiple sequence alignment with high accuracy and high throughput. *Nucleic Acids Res.* **2004**, *32*, 1792–1797.
2. Crooks, G.E.; Hon, G.; Chandonia, J.M.; Brenner, S.E. WebLogo: A sequence logo generator. *Genome Res.* **2004**, *14*, 1188–1190.
3. Numata, T.; Ishimatsu, I.; Kakuta, Y.; Tanaka, I.; Kimura, M. Crystal structure of archaeal ribonuclease P protein Ph1771p from Pyrococcus horikoshii OT3: an archaeal homolog of eukaryotic ribonuclease P protein Rpp29. *RNA* **2004**, *10*, 1423–1432.
4. Amero, C.D.; Boomershine, W.P.; Xu, Y.; Foster, M. Solution structure of Pyrococcus furiosus RPP21, a component of the archaeal RNase P holoenzyme, and interactions with its RPP29 protein partner. *Biochemistry* **2008**, *47*, 11704–11710.

© 2016 by the authors; licensee MDPI, Basel, Switzerland. This article is an open access article distributed under the terms and conditions of the Creative Commons by Attribution (CC-BY) license (http://creativecommons.org/licenses/by/4.0/).
